# Uncovering hidden cross-regional environmental risks: Network evidence from off-site penalties and implications for pollution transfer in China

**DOI:** 10.1016/j.isci.2026.116512

**Published:** 2026-07-02

**Authors:** Feng Hu, Huijie Yang, Shuang Zhao, Liping Qiu, Shaobin Wei, Jiahan Hu, Yufeng Chen, Hao Hu, Haiyan Zhou

**Affiliations:** 1 Institute of International Business & Economics Innovation and Governance, Shanghai University of International Business and Economics, Shanghai 200336, China; 2 International Business School, Shanghai University of International Business and Economics, Shanghai 201620, China; 3 College of Management, Ningbo University of Finance & Economics, Ningbo 315175, China; 4 CEEC Economic and Trade Cooperation Institute, Ningbo University, Ningbo 315211, China; 5 College of Engineering, University of Perpetual Help System Laguna, Biñan, Laguna 4024, Philippines; 6 School of Economics and Management, Zhejiang Normal University, Jinhua 321004, China; 7 School of Economics, Shanghai University, Shanghai 200444, China; 8 Graduate School, Nueva Ecija University of Science and Technology, Cabanatuan 3100, Philippines

**Keywords:** pollution transfer, environmental penalties, spatial network analysis, geographical detector, sustainable development, urban management

## Abstract

The pressures of economic development and environmental governance are growing increasingly intense. This study uses 2014–2023 off-site environmental penalty data of Chinese listed firms to construct weighted intercity networks of cross-regional environmental violation linkages and compares the spatial patterns between firms in polluting industries and those in other industries via social network analysis, spatial statistics, and geographical detector methods. The results show that cross-regional environmental violation linkages have become increasingly dense, with firms in other industries accounting for a fast-growing share of cross-city violations, and these linkages exhibit significant spatial clustering patterns. Specifically, levels of economic development, industrial structure, and financial capacity are positively and significantly associated with cities’ network centrality and intensity, while environmental regulation and public health factors show no significant associations. These findings contribute to the literature by offering a behavioral and outcome-based perspective on cross-regional environmental violations and highlighting the need for unified governance frameworks.

## Highlights

• Off-site penalties uncover cross-regional pollution risks in China
• Intercity networks capture pollution transfer linkages among firms
• Non-polluting firms show stronger and expanding network structures
• Economic and financial factors drive network evolution patterns among factors

## Graphical abstract

**Figure:**
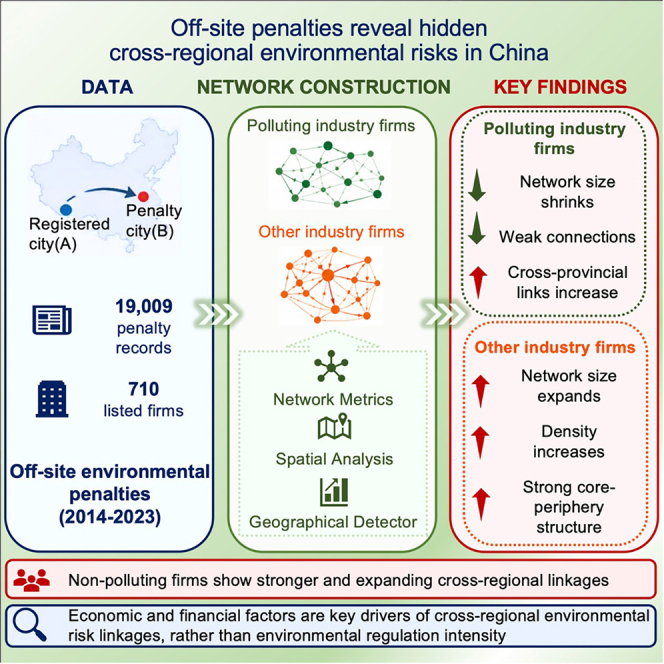

## Teaser

Environmental science; Environmental policy; Pollution

## Introduction

Since the launch of the reform and opening-up policy, China’s economy has grown rapidly, achieving remarkable accomplishments. However, this has also been accompanied by frequent occurrences of environmental pollution,^1^ particularly in the form of cross-provincial pollution transfer. For instance, several batches of typical environmental law enforcement cases released by the Ministry of Ecology and Environment of the People’s Republic of China (MEE) have reported incidents such as: *the case of Wang XX and others in Yongkang City, Jinhua, Zhejiang Province, illegally dumping and disposing of hazardous waste across provinces*; *the case of Shanghai Shengkuo Foundation Engineering Co., Ltd., illegally transferring construction slurry across provinces*. In most of these cases, pollution was transferred from cities with higher administrative ranks or higher levels of economic development to those with lower administrative ranks or relatively weaker economic development (https://www.mee.gov.cn/ywgz/sthjzf/zfzdyxzcf/202411/t20241119_1095678.shtml; https://www.mee.gov.cn/ywgz/sthjzf/zfzdyxzcf/202411/t20241125_1096570.shtml; https://www.mee.gov.cn/ywgz/sthjzf/zfzdyxzcf/202211/t20221118_1005261.shtml). To address environmental challenges, the state has enacted stringent laws and regulations, including *the Environmental Protection Law of the People’s Republic of China*, which have significantly improved ecological conditions and strongly promoted high-quality, sustainable economic development.^2^^,^^3^ Nevertheless, with growing national attention to pollution and the strengthening of local environmental regulations, some cities have begun to conduct spatially oriented pollution transfer under the guise of industrial relocation. Analyzing pollution transfer is highly important for promoting cross-regional pollution control and quality governance within urban networks.

Urban network research has shifted from global to regional and further to intra-urban scales.^4^^,^^5^^,^^6^^,^^7^^,^^8^^,^^9^^,^^10^^,^^11^ Based on modified gravity models, input-output models, and social network analysis (SNA), studies have employed macroeconomic data to reveal the basic features of intercity industrial networks;^12^^,^^13^ at the enterprise level, data on headquarters-subsidiary relations, investment ties, and business linkages have been used to map intercity corporate networks;^14^^,^^15^^,^^16^^,^^17^^,^^18^ at the infrastructure and innovation level, transportation networks, information exchange data, patent cooperation and transfer, and other related data have been adopted to construct intercity infrastructure and innovation networks.^19^^,^^20^^,^^21^^,^^22^^,^^23^^,^^24^ Moreover, methods such as QAP, exponential random graph models, and geographical detectors (Geodetector) have been applied to explore the influencing factors of networks, such as urban economic scale, innovation capacity, and multidimensional proximity.^25^^,^^26^^,^^27^^,^^28^^,^^29^^,^^30^

With respect to pollution transfer, existing studies focus on two main aspects: its causes and forms. External factors, such as environmental regulation and local government performance assessment, and internal factors, such as enterprises’ efforts to reduce compliance costs, are key drivers.^31^^,^^32^^,^^33^^,^^34^^,^^35^^,^^36^ In terms of forms, scholars have mainly examined explicit transfers, including cross-border investment and the relocation of polluting industries, enterprises, or factories.^37^^,^^38^^,^^39^^,^^40^^,^^41^ More recently, concealed forms, such as those associated with equity linkages, have attracted increasing attention.^42^^,^^43^^,^^44^^,^^45^ For example, Lu C. et al. and Wang Z. et al., using parent-subsidiary registration data in polluting industries, constructed urban pollution transfer networks and found that central cities play key roles, with “beggar-thy-neighbor” patterns more evident in eastern regions.^44^^,^^45^ The pollution haven hypothesis suggests that pollution shifts from regions with stringent regulations to those with looser ones.^46^ In contrast, the Porter hypothesis argues that well-designed regulations can enhance competitiveness and achieve win-win outcomes.^47^ However, their validity remains debated. For instance, Fu et al. found that polluting industries in China are relocating from eastern to central and western regions.^48^ At the same time, Grether et al. and Beghin et al. reported that developing countries do not necessarily become pollution havens.^49^^,^^50^

Research has demonstrated that environmental pollution has substantial negative effects on public health. Industrial emissions, heavy metal contamination of land, and pollutants such as PM_2.5_ significantly increase the incidence of respiratory diseases, cancers, and other health problems. Moreover, regions with relatively lower levels of governance capacity tend to face greater public health risks, so that pollution transfer has become a critical factor exacerbating health inequalities.^20^^,^^51^^,^^52^^,^^53^^,^^54^^,^^55^^,^^56^ A review of the literature reveals two main gaps. On the one hand, most existing studies infer pollution transfer pathways from ownership relationships between parent and subsidiary companies of polluting enterprises. However, the registration links do not necessarily correspond to actual pollution transfer. On the other hand, previous research has primarily focused on polluting firms, while overlooking that nonpolluting firms may also engage in pollution transfer during production and operational activities. Against this background, this study draws on off-site environmental penalty data from both polluting listed firms and other listed firms in China from 2014 to 2023. By applying SNA and the Geodetector method, we analyze the spatiotemporal evolution of cross-regional environmental violations in China and elucidate the underlying factors associated with this process. The core indicator measures the spatial association between a listed firm’s registered city and the city where penalties are issued, reflecting the cross-regional distribution of firms’ violation behaviors. However, this is a behavioral proxy for cross-regional environmental risks, not direct evidence of strategic pollution relocation or physical activities.

The contributions of this study are as follows.

(1) By constructing a network based on the off-site environmental penalty data of listed firms, we are able to capture the observed spatial linkages of environmental violations across regions, providing a behavioral proxy that is closely related to pollution transfer dynamics.
(2) By comparing polluting listed firms with other listed firms, we provide empirical evidence that the latter exhibit stronger and faster-growing cross-regional environmental violation linkages. This finding challenges the widely held assumption that industries outside the official polluting list carry inherently low environmental risk.^42^^,^^43^^,^^44^^,^^45^
(3) These findings enrich the understanding of intercity environmental linkages and provide an empirical foundation for designing more equitable and sustainable environmental governance mechanisms.

## Results

### Basic network properties

As shown in Table 1, both the polluting listed firm and other listed firms’ networks significantly increase in the number of participating cities as well as in the intercity transfer linkages, indicating that cross-regional environmental violations have become broader in scope and more complex in structure. However, the two types of networks demonstrate markedly different evolutionary trends.

**Table 1:** Attributes of China’s cross-regional environmental violation network, 2014–2023

Network attributes	Polluting listed firms		Others listed firms	
	2014–2018	2019–2023	2014–2018	2019–2023
Number of nodes	81	107	127	184
Number of edges	79	90	246	474
Average degree	0.975	0.841	1.937	2.576
Average weighted degree	2.821	1.229	5.657	5.745
Network diameter	8	5	8	10
Network density	0.012	0.008	0.015	0.014
Average clustering coefficient	0.012	0.014	0.061	0.070
Average path length	2.953	1.825	3.387	3.732

For the polluting network, connections remain sparse (increasing from 19 to 90), whereas average degree (0.975 → 0.841), network diameter (8 → 5), and average path length (2.953 → 1.825) all decrease. Although the number of cities involved in cross-regional environmental violations increased slightly (81 → 107), the overall linkage intensity weakened (2.821 → 1.229).

In contrast, the other listed firm’s network becomes significantly denser (with the number of edges increasing from 246 to 474). Indicators such as the average degree (1.937 → 2.576), network diameter (8 → 10), and average path length (3.387 → 3.732) all displayed increasing trends. Moreover, both the number of cities involved (127 → 184) and the linkage intensity (5.657 → 5.745) increased substantially.

These findings suggest that in the period following 2018, large-scale cross-regional environmental violations associated with polluting listed firms exhibited a contracting trend, whereas violation linkages among other listed firms became increasingly prominent. The concentration of large-scale violation cases coincides temporally with the backdrop of the continuous strengthening of environmental regulation intensity in China in recent years, which may be attributed to the increasing intensity of interregional environmental supervision and the rising costs of cross-regional emissions. Conversely, cross-regional environmental violations in other industries challenge conventional assumptions. For example, in accordance with environmental penalty data, Shenwu Energy Saving Co., Ltd., a firm outside of MEE’s polluting industry list, registered in Nanchang, Jiangxi Province, was administratively penalized by the Environmental Protection Bureau of Linghai City, Liaoning Province, for directly discharging untreated wastewater. While heavily polluting industries have long been the primary focus of environmental regulation, insufficient attention to the potential risks of other industries has led to relatively high transfer costs for firms in heavy polluting industries.

Furthermore, there are significant differences in the core-periphery structures of these two networks. For polluting listed firms, the average degree in both periods was below 1, and network density was extremely low (0.012 and 0.008, respectively), indicating that most city nodes were either isolated or connected through only a very small number of links. The average clustering coefficient consistently remained below 0.015, indicating virtually no triangular relationships and, consequently, the absence of interconnected core groups. These characteristics suggest that the networks of polluting listed firms lack a stable core-periphery structure. In contrast, for other listed firms, the average degree in both periods exceeded 1.9. Despite a substantial increase in the number of nodes, network density remained around 0.014. The average clustering coefficient (0.061 → 0.070) is five times that of the polluting listed firms’ network, indicating the presence of local clustering. These characteristics are consistent with a distinct “core-periphery” structure, wherein a small group of tightly connected core cities coexists with a larger peripheral region. This structure reveals distinct behavioral patterns among the two groups of firms, providing empirical evidence for the design of tiered, zoned, and categorized environmental governance policies.

### Cross-regional environmental violation networks of polluting listed firms

**Figure 1:**
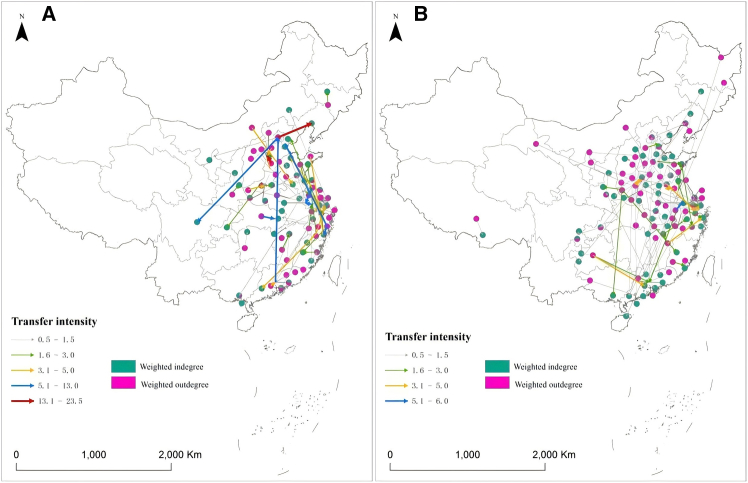
Cross-regional environmental violation network of polluting listed firms in Chinese cities, 2014–2023 This figure uses a combination of dots and lines to illustrate the pattern of cross-regional environmental violations by Chinese polluting listed firms between 2014 and 2023. (A) 2014–2018. (B) 2019–2023.

Based on the classification of the link strengths in Stage 1 into five levels, Figure 1 shows that in the polluting network, the link strength is significantly lower than that in the other firms’ network. The network structure is relatively unstable, and its core pathways change substantially over time.

In Stage 1, the Yangtze River Delta (YRD) and the Beijing-Tianjin-Hebei region were the primary urban agglomerations generating outward violation linkages, accounting for 20.13% and 14.22% of the total linkage intensity, respectively. In Stage 2, the Central Plains and the West Coast of the Taiwan Strait urban agglomerations became the main centers of outward linkage, accounting for 9.13% and 7.60%, respectively. Unlike previous studies, our findings indicate that the eastern region is both a major origin and destination of cross-regional environmental violations. Although the absolute linkage intensity into the eastern, central, and western regions has declined, their proportional shares have increased, whereas the opposite trend is observed in the northeast region.

No cross-regional environmental violation linkages occur between high-tier cities. The share of linkage from high-tier cities to low-tier cities decreased from 14.88% in 2014–2018 to 5.70% in 2019–2023. Moreover, the share of linkage from low-tier cities to high-tier cities and to other low-tier cities increased from 11.60% to 73.52%–12.55% and 81.75%, respectively. Thus, cross-regional environmental violation linkages predominantly occur between low-tier cities, while listed firms registered in higher-ranking cities are mostly penalized in lower-ranking cities.

In terms of cross-provincial penalties, both the number of penalties and their intensity increased (from 41 cases and 115.5 to 59 cases and 90), with their shares rising from 51.9% to 50.55%–65.56% and 68.44%, respectively. This suggests that cross-provincial linkages have gradually become more prominent for polluting listed firms, while the constraining role of administrative boundaries appears to be weakening. Intraprovincial cases may be subject to more direct provincial-level regulatory pressure and greater information transparency, whereas cross-provincial activities may be associated with differences in regulatory environments, information asymmetry, and coordination costs, which is consistent with the pollution haven hypothesis.

The top ten cities with negative net out-degree from 2014 to 2018 were Shijiazhuang, Panjin, Nanjing, Wenzhou, Tianjin, Wuhan, Chengdu, Sanming, Suzhou, and Lianyungang. From 2019 to 2023, the top ten cities were Shanghai, Guangzhou, Ningbo, Zhenjiang, Zhengzhou, Foshan, Xiamen, Xinxiang, Suzhou, and Sanming. The number of higher-tier cities decreased from five to four, with violation flows shifting primarily toward the eastern coastal regions. This trend indicates that under strict industry-specific environmental supervision, high-tier cities are gradually shedding their role as environmental risk bearers, while core cities in central and western China have completely dropped out of the top 10 list of risk bearers. Notably, only 2 cities ranked in the top 10 across both periods, reflecting the extremely low stability of risk-bearing roles. Moreover, negative externalities are more pronounced in higher-tier cities. A comparison of the average net out-degree of municipalities directly under the central government, subprovincial cities, provincial capitals, and other prefecture-level cities (Table 2) reveals that higher-tier cities generally experience a net inflow of cross-regional environmental violations, while other prefecture-level cities exhibit a net outflow. This finding indicates that the network externalities of polluting listed firms show a pronounced tendency toward higher-tier cities. This may be because the headquarters of these polluting listed firms are constrained by policies to locate in lower-tier cities, yet their operational activities necessitate conducting business in higher-tier cities, thereby generating violations across regions.

**Table 2:** Average centrality of cross-regional environmental violation networks for polluting listed firms in different city types

Type		Municipalities directly under central government	Subprovincial cities	Provincial capitals	Others prefecture-level cities
2014–2018	Average out-degree	8.500	1.308	2.522	1.562
	Average in-degree	6.625	4.385	5.152	0.988
	Average net out-degree	1.875	−3.077	−2.630	0.573
2019–2023	Average out-degree	1.875	0.615	1.109	0.940
	Average in-degree	4.125	2.346	1.826	0.702
	Average net out-degree	−2.25	−1.731	−0.717	0.239

Note: Net out-degree is defined as the difference between a city’s outgoing and incoming linkages, calculated as out-degree minus in-degree.

On the basis of the top 20 cities for cross-regional environmental violations by China’s polluting listed companies from 2014 to 2018, a comparison of the changes from 2019 to 2023 reveals a very weak path dependency in these patterns (Table 3). Nearly all the core city pairs from 2014 to 2018 experienced decreases in their linkage intensity to zero or near-zero levels during 2019–2023. For instance, linkages from Xingtai to Shijiazhuang decreased from 16 to 0, while those from Beijing to Panjin decreased from 23.5 to 0.5. The prevalence of negative growth rates indicates extremely low path dependency in cross-regional environmental violations by listed polluting firms. This pattern is observed alongside strengthened local environmental regulation and higher environmental compliance costs, with a concurrent reduction in cross-city environmental violation linkages among polluting listed firms.

**Table 3:** Correlations between cross-regional environmental violation cities by polluting listed firms

Rank	City pairs	2014–2018	2019–2023	Growth rate	Rank	City pairs	2014–2018	2019–2023	Growth rate
1	Beijing→Panjin	23.5	0.5	−23	12	Huzhou→Suzhou	4.5	0	−4.5
2	Xingtai→Shijiazhuang	16	0	−16	13	Yantai→Wenzhou	4.5	0	−4.5
3	Taizhou (Zhejiang)→Wenzhou	13	0	−13	14	Yancheng→Binzhou	4.5	0	−4.5
4	Changzhou→Nanjing	8.5	0	−8.5	15	Baoding→Shijiazhuang	4	0	−4
5	Guangzhou→Beijing	8.5	0	−8.5	16	Foshan→Guangzhou	4	0	−4
6	Beijing→Chengdu	7.5	0	−7.5	17	Jiaxing→Lianyungang	4	0	−4
7	Jingmen→Wuhan	7.5	0	−7.5	18	Ulanqab→Xuzhou	4	0.5	−3.5
8	Taizhou (Zhejiang)→Tianjin	7.5	0	−7.5	19	Yangzhou→Nanjing	4	0	−4
9	Yancheng→Nanjing	7.5	0	−7.5	20	Lishui→Shanghai	3.5	0	−3.5
10	Handan→Shijiazhuang	5	0	−5	21	Lishui→Yunfu	3.5	0	−3.5
11	Sanmenxia→Zhengzhou	5	0	−5	–	–	–	–	–

ArcGIS software was used to calculate global Moran’s I for the out-degree and in-degree of cross-regional environmental violation networks among listed polluting firms (Table 4), revealing that the Moran’s I values are significantly positive and show a slight upward trend. This finding indicates that during 2014–2023, cities with net outward violation linkages and cities with net inward violation linkages both exhibited a pronounced positive spatial clustering pattern overall. In other words, cities with high/low tiers of cross-regional environmental violations are spatially adjacent to one another and are not completely isolated. Such violations are spatially associated with neighboring cities while simultaneously reflecting broader regional interaction patterns.

**Table 4:** Moran’s I out-degree and in-degree for cross-regional environmental violation networks of polluting listed firms

Type		In-degree	Out-degree
2014–2018	Moran’s I	0.029	0.052
	Z value	3.095	4.246
	*p* value	0.002	0.000
2019–2023	Moran’s I	0.093	0.078
	Z value	7.031	5.877
	*p* value	0.000	0.000

Further spatial hotspot detection was employed, utilizing the Getis–Ord Gi∗ index and natural breakpoint method to classify areas into cold spots, subcold spots, subhot spots, and hot spots. The findings reveal that the YRD urban cluster (including those of Nantong, Suzhou, Shanghai, Hangzhou, and so forth) remained a hot spot in both Stage 1 and Stage 2, indicating that this region serves as a major concentration area for cross-regional environmental violations, with pronounced spatial agglomeration effects. A few cities (e.g., Taizhou (Jiangsu) and Lianyungang) exhibited subhotspot status in terms of inward linkage intensity but maintained a hotspot status in terms of outward linkage intensity, indicating relatively active outward linkages alongside comparatively weaker inward linkages. Cold zones appeared primarily on the periphery of the Pearl River Delta (Shanwei, Huizhou), southwestern regions (Wuzhou, Maoming), and remote areas (Sansha, Ali Prefecture), with relatively few instances. Thus, the network is highly concentrated along the eastern coast, with low participation from central and western regions. Overall, high-value linkage areas are continuously strengthening, while cold spots are becoming more scattered. The hotspot patterns of most cities remained stable between 2014 and 2023.

### Cross-regional environmental violation network of other listed firms

**Figure 2:**
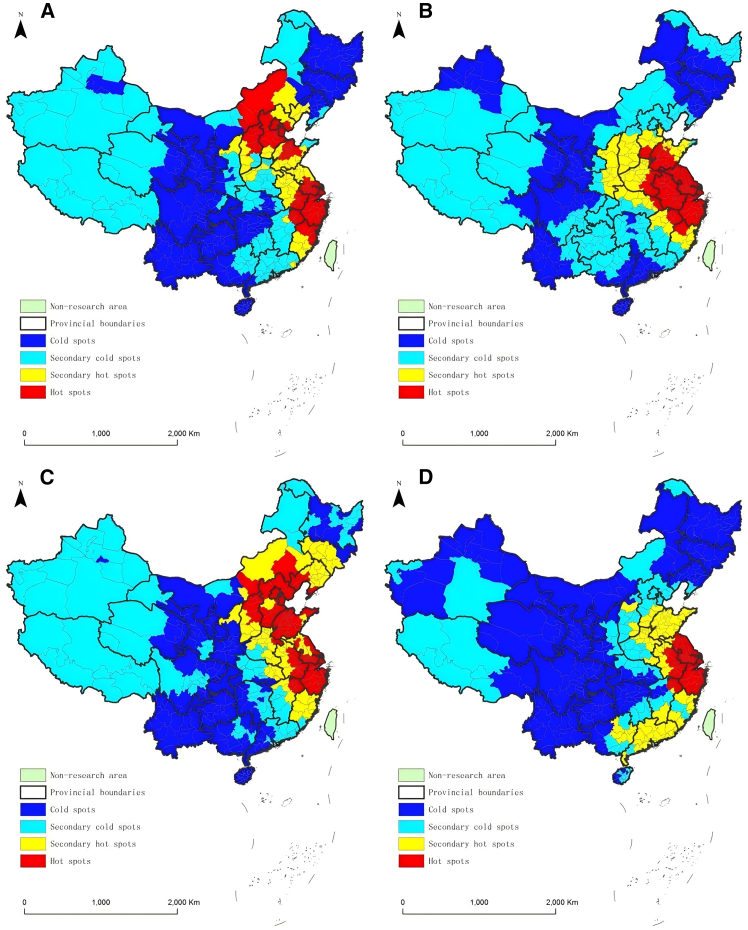
Spatial distribution of hotspots and coldspots in urban cross-regional environmental violations by polluting listed firms in China, 2014–2023 This figure depicts the spatial distribution of hotspots and coldspots for cross-regional environmental violations by polluting listed firms across Chinese cities from 2014 to 2023, covering both outward off-site penalties (A and B) and inward off-site penalties (C and D) in two periods. (A) Outward Off-site Penalties (2014–2018). (B) Outward Off-site Penalties (2019–2023). (C) Inward Off-site Penalties (2014–2018). (D) Inward Off-site Penalties (2019–2023).

As shown in Figure 2, the cross-regional environmental violation network of other listed firms exhibits higher linkage intensity and a larger scale.

In Stage 1, the YRD and the Beijing-Tianjin-Hebei region were the primary urban agglomerations generating outward cross-regional environmental violation linkages, accounting for 18.93% and 26.10% of the total linkage intensity, respectively. In Stage 2, the YRD and the Guanzhong urban agglomeration became the main centers of outward linkages, accounting for 14.43% and 8.37%, respectively. Unlike previous studies, our findings indicate that the eastern region serves as both a major origin and destination of cross-regional environmental violations. Although the intensity of inward linkages in the eastern, central, and western regions increased substantially, only the proportion in the central region tended to increase, whereas the opposite trend was observed in the northeast region.

The share of linkages from high-tier cities to high-tier cities fell from 2.09% to 0.76%, and to low-tier cities from 24.22% to 5.72%. Conversely, the share of linkages from low-tier cities to high- and low-tier cities increased from 7.65% to 66.04%–8.85% and 84.67%, respectively. This finding indicates that cross-regional environmental violations primarily occur among low-tier cities, while linkages from high-tier cities are more likely to be directed toward low-tier cities.

In terms of cross-provincial patterns, both the number of cases and their linkage intensity increased from 176 to 534 to 326 and 662.5, respectively, while their proportional shares decreased from 71.54% and 74.32%–62.68% and 68.78%, respectively. This suggests that cross-provincial linkages remain an important pattern among other listed firms, while intraprovincial cases have gradually increased, indicating that the effect of administrative boundaries is becoming more pronounced.

**Figure 3:**
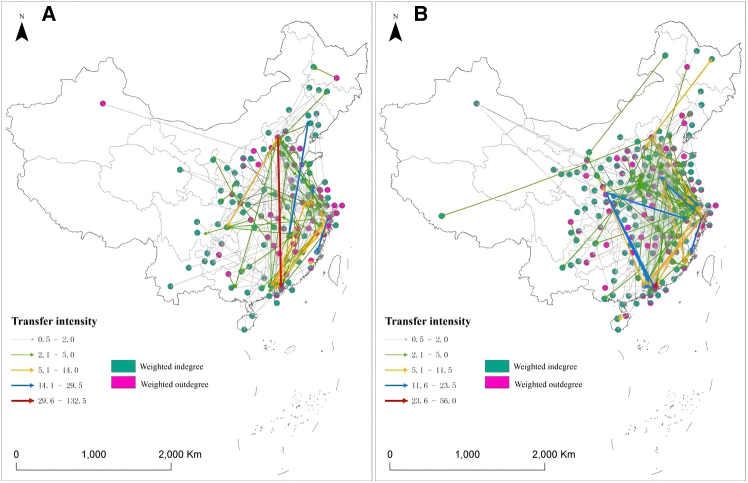
Cross-regional environmental violation network of other listed firms in Chinese cities, 2014–2023 This figure uses a combination of dots and lines to illustrate the pattern of cross-regional environmental violations by other listed firms of China between 2014 and 2023. (A) 2014–2018. (B) 2019–2023.

In the other listed firms’ network (Figure 3), the top ten cities with the most negative net out-degree in 2014–2018 were Shenzhen, Huizhou, Fuzhou, Jinzhou, Nanjing, Foshan, Yancheng, Chongqing, Sanming, and Qingdao. In 2019–2023, the top ten cities were Huizhou, Shanghai, Foshan, Wuxi, Fuzhou, Guangzhou, Nanjing, Suzhou, Jinan, and Qingdao. The number of high-tier cities increased from five to six, the spatial distribution of cross-regional environmental violation flows shifted primarily toward the eastern coastal regions, and environmental externalities associated with these activities became more pronounced in high-tier cities. In stark contrast to the other network, high-tier cities in this network have evolved into dual cores of both risk outflow and inflow. Despite the persistent net outflow of violations, their share in the top 10 recipient locations has continued to rise. The spatial pattern has solidified into a stable core-satellite agglomeration centered on eastern coastal megacities. Four overlapping cities across the two periods exhibit strong path dependence in their risk-bearing roles, forming a fixed division of labor between core cities and their surrounding satellite cities.

A comparison of the average net out-degree across different city ranks (Table 5) revealed that the average net out-degree of high-tier cities decreased from 10.489 in 2014–2018 to 1.039 in 2019–2023, whereas that of other prefecture-level cities decreased from 3.088 to −0.901 over the same period. This finding indicates that high-tier cities largely maintained stronger outward linkages, whereas low-tier cities exhibited the opposite pattern. The negative externalities in the other listed firms’ network tend to be concentrated in low-tier cities. High-tier cities face stricter environmental regulations and higher land and labor costs, which incentivize firms to relocate production bases and reduce resistance to transferring production. In contrast, low-tier cities face substantial development pressures, and their economic growth priorities may temporarily outweigh environmental considerations, which may be associated with higher levels of inward linkages.

**Table 5:** Average centrality of cross-regional environmental violation networks for other listed firms in different types of cities

Type		Municipalities directly under central government	Subprovincial cities	Provincial capitals	Others prefecture-level cities
2014–2018	Average out-degree	47.250	13.700	11.083	1.544
	Average in-degree	17.500	15.833	7.233	2.047
	Average net out-degree	29.750	−2.133	3.850	0.503
2019–2023	Average out-degree	17.125	22.767	11.917	3.088
	Average in-degree	25.375	12.600	10.716	3.988
	Average net out-degree	−8.250	10.167	1.200	−0.901

Note: Net out-degree is defined as the difference between a city’s outgoing and incoming linkages, calculated as out-degree minus in-degree.

A comparison of the top 20 cities for cross-regional environmental violations by other listed firms in China from 2014 to 2018 (Table 6) reveals that, compared with polluting listed firms, other listed firms exhibit relatively lower path dependency in these patterns from 2019 to 2023. Among the top 20 city pairs from 2014 to 2018, most experienced significant declines or near disappearance of linkage intensity between 2019 and 2023. For instance, the linkage intensity from Beijing to Shenzhen decreased from 132.5 to 1.5, and Nanchang→Jinzhou decreased from 29.5 to 0. Only a few, such as Shenzhen→Huizhou (from 25.5 to 56), showed strengthened linkages, indicating that other listed firms tend to shift away from previously dominant high-intensity linkage patterns rather than maintaining persistent structures.

**Table 6:** Correlations between cross-regional environmental violation cities by other listed firms

Rank	City pairs	2014–2018	2019–2023	Growth rate	Rank	City pairs	2014–2018	2019–2023	Growth rate
1	Beijing→Shenzhen	132.5	1.5	−131	14	Ningbo→Zhongshan	6.5	0	−6.5
2	Nanchang→Jinzhou	29.5	0	−29.5	15	Taizhou (Zhejiang)→Wenzhou	6.5	0	−6.5
3	Ningbo→Fuzhou	29	16	−13	16	Guangzhou→Nanjing	6	0	−6
4	Shenzhen→Huizhou	25.5	56	30.5	17	Quanzhou→Xiamen	6	0	−6
5	Taizhou (Jiangsu)→Yancheng	18	5	−13	18	Wuxi→Nanjing	6	1	−5
6	Shangrao→Guangzhou	14	1.5	−12.5	19	Baoding→Foshan	5	0	−5
7	Taizhou (Zhejiang)→Sanming	11.5	0	−11.5	20	Baoding→Cangzhou	4.5	0	−4.5
8	Ningbo→Foshan	10	6	−4	20	Baoding→Shijiazhuang	4.5	2	−2.5
9	Beijing→Chongqing	9	0	−9	20	Harbin→Qiqihar	4.5	0	−4.5
10	Changzhou→Yancheng	8	2.5	−5.5	20	Nanchang→Chongqing	4.5	2	−2.5
11	Shenzhen→Beijing	7.5	1.5	−6	20	Ningbo→Jiangmen	4.5	1	−3.5
12	Wenzhou→Jiaxing	7.5	0	−7.5	20	Ningbo→Xuzhou	4.5	0	−4.5
13	Baoding→Beijing	6.5	0	−6.5	20	Shangrao→Fuyang	4.5	1.5	−3

Moran’s I for the out-degree and in-degree of the cross-regional environmental violation networks among other listed firms (Table 7) reveals that both Moran’s I values are significantly positive. Notably, Moran’s I for cities with high outward linkages shows a marked decline, while that for cities with high inward linkages exhibits the opposite trend. This finding indicates that during the 2014–2023 period, cities with high and low levels of cross-regional environmental violations demonstrated a pronounced positive clustering pattern. That is, cities with high and low levels of linkages are spatially adjacent to one another and are not entirely spatially isolated. Such violations exhibit clear spatial association patterns across neighboring cities, reflecting interconnected regional structures rather than independent distributions. At the same time, this positive clustering pattern is more pronounced among cities with higher levels of inward linkages. Cities with relatively low levels of cross-regional environmental violations also tend to be geographically proximate rather than completely independent. These spatial patterns suggest that cross-regional environmental violations are embedded within broader regional interaction contexts and display varying degrees of spatial concentration. Notably, this positive clustering trend becomes more evident among cities with higher inward linkage intensity, while it is comparatively weaker among cities characterized by outward linkage dominance.

**Table 7:** Moran’s I of outflow and inflow for the cross-regional environmental violation networks of other listed firms

Type		In-degree	Out-degree
2014–2018	Moran’s I	0.063	0.303
	Z value	6.223	3.102
	*p* value	0.000	0.002
2019–2023	Moran’s I	0.209	0.080
	Z value	15.465	6.149
	*p* value	0.000	0.000

**Figure 4:**
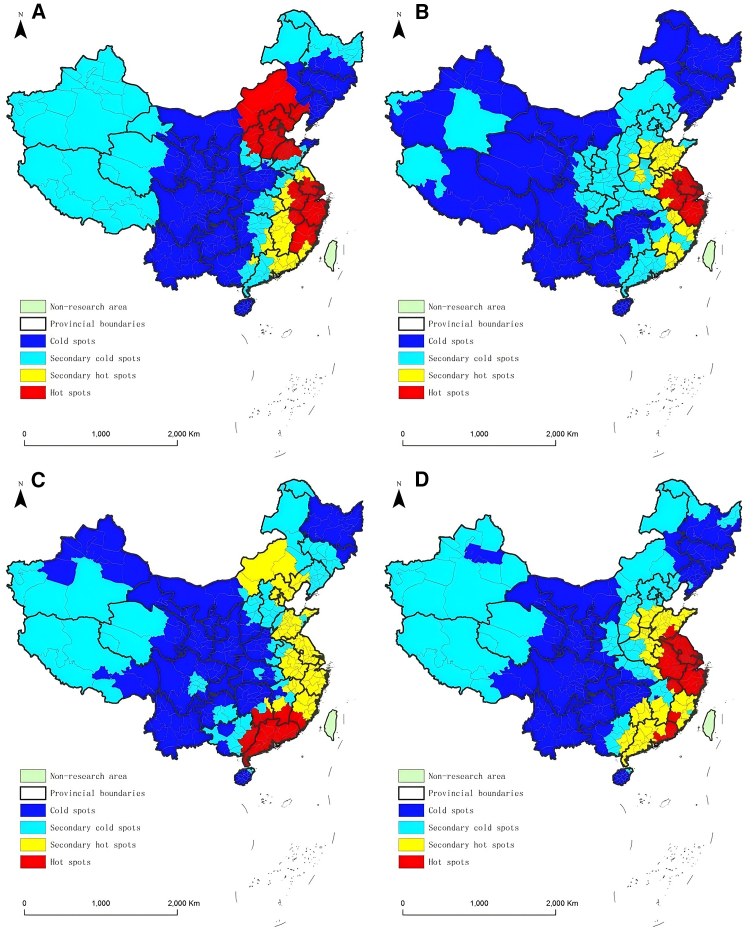
Spatial distribution of hotspots and coldspots in urban cross-regional environmental violations by other listed firms in China, 2014–2023 This figure depicts the spatial distribution of hotspots and coldspots for cross-regional environmental violations by other listed firms across Chinese cities from 2014 to 2023, covering both outward off-site penalties (A and B) and inward off-site penalties (C and D) in two periods. (A) Outward Off-site Penalties (2014–2018). (B) Outward Off-site Penalties (2019–2023). (C) Inward Off-site Penalties (2014–2018). (D) Inward Off-site Penalties (2019–2023).

Further spatial hotspot detection (Figure 4) was employed, utilizing the Getis–Ord Gi∗ index and the natural break method to classify areas into cold spots, subcold spots, subhot spots, and hot spots. The vast majority of cities remained cold spots or subcold spots throughout the 2014–2023 period. In contrast, economically vibrant regions, such as the YRD, Pearl River Delta, and coastal Fujian, exhibited hotspot clusters in terms of both inward and outward linkages, with these clusters progressively expanding toward central China. These hotspot areas show strong bidirectional linkage characteristics, gradually forming an unbalanced spatial pattern characterized by “hot in the east, cold in the west.” This spatial pattern is likely driven by a combination of regional industrial division of labor, differences in environmental regulations, and corporate strategic planning.

### Driving factors of urban pollution transfer in China

#### Variable selection

A synthesis of previous scholarly research reveals that urban environmental policies, economic development, public health, financial scale, and innovation and infrastructure significantly influence cross-regional environmental violations.^32^^,^^33^^,^^34^^,^^35^^,^^36^^,^^37^^,^^38^^,^^39^^,^^40^^,^^41^^,^^42^^,^^43^^,^^44^^,^^45^^,^^46^^,^^47^^,^^48^^,^^49^^,^^50^ Given this, this study employs weighted degree centrality, weighted in-degree, and weighted out-degree of the 2019–2023 network as explanatory variables. The indicators representing the economic development dimension are total retail sales of consumer goods, shares of secondary and tertiary industries in GDP, and GDP per capita; those representing the public health dimension are the number of physicians per 10,000 people and the number of hospital beds per 10,000 people; those representing the financial scale dimension are year-end balance of CNY deposits in financial institutions and the year-end balance of CNY loans in financial institutions; and the indicators representing the innovation and infrastructure dimension are telecommunications and postal service revenue, education expenditure, science and technology expenditure, and the number of patent authorizations; these are the dependent variables. Environmental regulation intensity was measured in Python by scraping the proportion of words related to low-carbon, PM_2.5_, sulfur dioxide, and so forth, in municipal government work reports relative to the total word count of the reports. This indicator captures the salience of environmental policy attention and policy orientation at the city level, rather than direct enforcement stringency. It should be noted that this proxy does not fully reflect regulatory enforcement intensity, inspection frequency, or penalty severity, which may be more directly relevant to firm behavior, particularly for listed firms operating across jurisdictions. Other influencing factors were sourced from the China Urban Statistical Yearbook.

#### Analysis results

Using the Geodetector method to analyze the city-level cross-regional environmental violation networks of polluting and other listed firms, we can identify the key driving factors influencing these violations (Table 8):

**Table 8:** Geodetector analysis results

Impact dimension	Impact factor	Polluting network			Others listed firms’ network		
		Weighted degree centrality	Weighted in-degree	Weighted out-degree	Weighted degree centrality	Weighted in-degree	Weighted out-degree
Environmental policy	strength of environmental regulations	0.024	0.010	0.039	0.016	0.013	0.026
Economic development	GDP per capita	0.152∗∗	0.190∗∗∗	0.048	0.296∗∗∗	0.240∗∗∗	0.204∗∗∗
	share of secondary and tertiary industries in GDP	0.115∗∗	0.114∗∗	0.033	0.212∗∗∗	0.140∗∗∗	0.104∗∗∗
	total retail sales of consumer goods	0.151∗	0.147∗	0.092	0.368∗∗∗	0.295∗∗∗	0.182∗∗∗
Public health	number of physicians per 10,000 people	0.039	0.021	0.041	0.048	0.031	0.031
	number of hospital beds per 10,000 people	0.021	0.060	0.063	0.006	0.020	0.003
Financial scale	year-end balance of CNY deposits in financial institutions	0.200∗	0.246∗∗	0.050	0.342∗∗∗	0.259∗∗∗	0.298∗∗
	year-end balance of CNY loans in financial institutions	0.196∗	0.247∗∗	0.070	0.399∗∗∗	0.295∗∗∗	0.313∗∗∗
Innovation and infrastructure	revenue from telecommunications and postal services	0.201	0.319∗	0.014	0.417∗∗∗	0.366∗∗∗	0.247∗∗∗
	education expenditures	0.200∗	0.255∗∗	0.066	0.392∗∗∗	0.311∗∗∗	0.245∗∗∗
	science and technology expenditures	0.169∗∗	0.198∗∗	0.110∗	0.424∗∗∗	0.347∗∗∗	0.305∗∗∗
	number of patents granted	0.149∗	0.274∗∗∗	0.023	0.430∗∗∗	0.364∗∗∗	0.326∗∗∗

Note: ∗*p* < 0.10, ∗∗*p* < 0.05, and ∗∗∗*p* < 0.01.

In the cross-regional environmental violation network of polluting listed firms, economic development and financial scale are the most significant factors associated with cities’ network positions among listed firms. Among the economic development indicators, per capita GDP is positively associated with weighted degree centrality, indicating that economically developed cities tend to exhibit higher levels of inward cross-regional environmental violation linkages, which may be related to differences in industrial capacity or resource absorption capability. The share of secondary and tertiary industries in GDP and total retail sales of consumer goods is also significantly associated with weighted degree centrality and in-degree, suggesting that economic structure and consumption vitality may be correlated with variations in cross-regional environmental violations. Within the financial dimension, deposit and loan balances are significantly associated with weighted in-degree, indicating that cities with abundant financial resources tend to exhibit higher levels of inward linkages, although the underlying mechanisms cannot be directly identified. Notably, weighted out-degree generally does not show strong or consistent associations with most explanatory variables (e.g., secondary industry share), showing weak correlations, suggesting that cross-regional environmental violations behavior may be influenced by more complex, non-economic factors.

In contrast, the network of other listed firms exhibits broader associations with economic and technological factors. All economic development indicators are significantly associated with weighted degree centrality, in-degree, and out-degree, suggesting that economically developed cities tend to occupy more central positions and exhibit both higher inward and outward linkages in this network. Financial scale, innovation, and infrastructure indicators are significantly associated with weighted degree centrality and out-degree, particularly innovation indicators, indicating that higher levels of technological innovation tend to coincide with higher levels of cross-regional environmental violation activities. Compared with the other listed firm’s network, the corresponding associations in the polluting network are generally weaker and less consistent, indicating potential differences in underlying patterns across the two types of firms.

Environmental policy and public health factors are not significantly associated with the network metrics in either network, which represents a notable empirical pattern. Environmental regulation intensity does not show statistically significant associations with network metrics in either network under the current specification, which may reflect that the relationship between environmental regulation and cross-regional environmental violations is complex. This proxy primarily reflects the level of policy attention rather than actual enforcement intensity. While the indicator shows no statistical significance, this does not imply that environmental policies have no effects, and their mechanisms or pathways of action require further identification. Similarly, public health status is not significantly associated with network position. The descriptive patterns suggest that cross-regional environmental violation linkages tend to occur between cities with different network rankings, and that lower-ranking cities tend to have relatively lower public health levels, suggesting that public health does not appear to be strongly associated with firms’ location choices in this context, although it may still be relevant in terms of the consequences of pollution exposure rather than location-selection mechanisms.

Overall, cities’ roles in cross-regional environmental violation networks are primarily associated with their levels of economic development, financial resource allocation, and technological innovation capacity, with other listed firms showing stronger associations with these factors than polluting listed firms. Environmental regulation intensity does not show the expected constraining association in either network, while public health resources do not show significant associations with network position. The underlying patterns differ fundamentally: Polluting listed firms show relatively stronger associations with financial factors, whereas other listed firms show stronger associations with economic and technological conditions.

## Discussion

### Conclusions

Drawing on off-site environmental penalty data for polluting and other listed firms, this study overcomes the limitations of relying solely on parent-subsidiary relations or heavily polluting industries. Using SNA, spatial statistics, and the Geodetector method, the spatial evolution patterns and driving mechanisms of cross-regional environmental violations in China are revealed. The main findings are as follows.

1. For polluting listed firms, the network has contracted, become looser, and shown more cross-provincial linkages, while the YRD remains a stable hotspot. For other listed firms, violation intensity has surpassed that of polluting firms and continues to grow, with a hotspot cluster expanding from the eastern coast inland and intra-provincial linkages increasing. These patterns reflect off-site violation behaviors rather than the strategic relocation of polluting production capacity studied in the pollution haven literature; thus, they do not contradict but instead extend existing knowledge by focusing on a different type of cross-regional environmental activity.
2. Both polluting and other listed firms exhibit cross-regional environmental violation linkages between high-tiers and low-tiers cities, with more than 80% occurring among low-tiers cities; in other listed firms, the negative externalities of high-tiers cities are amplified.
3. Cities’ roles in the cross-regional environmental violation network are driven mainly by their level of economic development, financial resource allocation, and technological innovation capacity, with other listed firms showing greater sensitivity to these factors than polluting listed firms.

### Implications for policy

Our findings reveal a core environmental justice concern that cross-regional environmental violations are significantly and disproportionately associated with interactions between high-tier and low-tier cities and a clear mismatch between environmental risk exposure and local health service capacity. Environmental risks are more likely to be borne by low-tier cities with relatively weak medical systems, which may exacerbate population health risks and regional health inequalities. Based on the observed risk-resource mismatch, this study puts forward the following policy recommendations.

1. Establishing Coordinated Health Governance and Compensation Mechanisms.

Establishing a cross-regional environmental health governance framework for cities with high outward and inward violation linkages is recommended. This framework can facilitate the flow of medical resources to areas receiving environmental violations, helping them enhance their risk response capacity. In addition, mechanisms for cross-jurisdictional environmental litigation and health impact compensation can be streamlined to assist affected communities in claiming legitimate compensation from enterprises responsible for cross-regional environmental violations.

2. Implementing a Health-Centered Monitoring and Accountability System.

Construction of an integrated monitoring system that links cross-regional environmental violations with potential public health impacts is suggested. This system can mandate health impact assessments for communities affected by relevant environmental risks, and the incorporation of a health restoration surcharge into environmental administrative penalties could be considered to internalize potential health costs. The surcharge can include estimated public health costs in the penalty amount, thereby increasing the economic costs of enterprises' high-risk operational activities and creating a stronger financial deterrent.

### Theoretical contributions

Extending measurement approaches to cross-regional environmental risk linkages: Existing studies often rely on parent-subsidiary relationships (e.g., headquarters-branch structures) to infer potential pathways for pollution transfer. This study complements that strand of literature by constructing a network from observable cross-regional environmental violation records, providing an alternative, data-driven perspective on firm-related environmental risk linkages. Rather than establishing direct pollution transfer mechanisms, this approach offers a descriptive and network-based representation of how environmental risks are spatially associated across regions.^42^^,^^43^^,^^44^^,^^45^

Broadening the empirical scope beyond traditionally defined polluting sectors: While prior research primarily focuses on firms in officially classified polluting industries, this study includes a wider set of listed firms to examine cross-regional environmental violation patterns. The findings suggest that firms outside traditionally defined polluting sectors also exhibit notable levels of cross-regional environmental risk linkages. This result does not necessarily imply environmentally motivated relocation behavior, but instead highlights the importance of considering firm network expansion and operational complexity when interpreting environmental risk patterns. In this sense, the study provides an empirical extension to existing discussions, rather than a revision of established theories such as the Pollution Haven Hypothesis.^42^^,^^43^^,^^44^^,^^45^

### Limitations of the study

This study focuses only on listed firms, while SMEs and foreign-invested enterprises require further investigation. Future research can also differentiate by pollution type (e.g., air, water, solid waste) to reveal distinct driving mechanisms and public health risks.

Off-site environmental penalties serve as a proxy but do not directly identify “pollution transfer” in the strict sense, which lacks actual business premises. Moreover, penalty amounts are subject to regional enforcement discretion and firms’ bargaining power, making them imperfect proxies for pollution severity. And the measurement of environmental regulation relies on a text-based proxy rather than actual enforcement intensity. Future work should incorporate litigation records, violation-based severity classification, and alternative regulatory measures (e.g., pollution tax intensity, inspection frequency).

This study uses only the factor detector module of Geodetector without interaction analysis, does not address network endogeneity or adopt QAP/ERGM methods, and relies solely on city-level macro data, omitting micro-level firm attributes such as parent-subsidiary relationships and corporate strategies. Future research can improve the analytical framework by combining advanced methods and micro-level data.

Over 90% of environmental violations occur within the same city, yet this study focuses solely on cross-regional linkages. Future research could explore intra-city spatial patterns (e.g., shifts from central to suburban areas) and conduct heterogeneity analyses by region, city tier, or regulatory intensity.

## Resource availability

### Lead contact

Further information and requests for resources should be directed to and will be fulfilled by the lead contact, Jiahan Hu (allenjiahanhu@outlook.com).

### Materials availability

This study did not generate new unique reagents.

### Data and code availability

• Data: Data reported in this paper will be shared by the lead contact upon request.
• Code: This paper does not report original code.
• Other items: Any additional information required to reanalyze the data reported in this paper are available from the lead contact upon request.

## Acknowledgments

This work was supported by the 10.13039/100014718National Natural Science Foundation of China (grant no. 72373135) and the 10.13039/501100013139Humanities and Social Sciences Research Fund of the Ministry of Education of China (grant no. 22YJAZH027).

## Author contributions

Conceptualization, funding acquisition, writing – original draft: F.H. writing – review and editing: H.J.Y. and H.Y.Z. validation: H.J.Y. data curation: S.Z. resources: L.P.Q. software, visualization: S.B.W. investigation, formal analysis: J.H.H. supervision, methodology: Y.F.C. project administration: H.H.

## Declaration of interests

The authors declare no competing interests.

## STAR★Methods

### Key resources table

**Table:** 

REAGENT or RESOURCE	SOURCE	IDENTIFIER
**Software and algorithms**		

Gephi	Gephi Consortium	Version 0.10.1; https://gephi.org
ArcGIS	Environmental Systems Research Institute	Version 10.8; https://www.arcgis.com/index.html
Microsoft Excel	Microsoft	Version 2021; https://www.microsoft.com
Geodetector	Chinese Academy of Sciences	http://geodetector.cn

**Other**		

Any additional information required to reanalyze the data reported in this paper	Available by request from lead contact	N/A

### Method details

#### Data sources

The data used in this study were obtained from the *Qixin Huiyan* database (https://b.qixin.com/ent/search/advanced). We extracted 19,581 records of environmental penalties imposed on listed companies from the “risk characteristics” section of the database. An off-site environmental penalty is defined as a case in which a firm registered at Location A is penalized at Location B, forming a cross-regional linkage between A and B.

First, we removed records missing a penalty year or penalizing authority, yielding 19,009 valid records (2014–2023) involving 710 listed firms. Over the sample period, the annual number of penalties exhibited a non-monotonic trend. The number of cases surged sharply in the early stage, with year-on-year growth rates of approximately 80% and 104% in 2016 and 2017, respectively. Followed by a decline phase, with year-on-year drops of about 28% and 19% in 2018 and 2019. Then shifted into a relatively stable period with slight fluctuations, with year-on-year increases of around 1% and 5% in 2020 and 2021, and saw another notable decrease, with a year-on-year drop of approximately 36% in 2022 and 2023. Second, using the batch query function, we retrieved each firm’s registered location, industry classification, the administrative city, and the province of the penalizing authority. Third, we weighted each penalty record according to the penalty amount: penalties of 0, 0.01–0.99, 1–9.99, 10–19.99, 20–49.99, 50–99.99, and above 100 (10,000 CNY) were assigned weights of 0.5, 1, 1.5, 2, 3, 4, and 5, respectively.^40^^,^^57^ This scheme measures the relative severity of violations, not the absolute pollution scale. Core spatiotemporal and structural network patterns are highly consistent with those from unweighted penalty counts, introducing no systematic bias to our core findings. Since the legal effect of penalty decisions confirms the existence of environmental violations, even zero-monetary penalties (e.g., warnings) reflect official recognition of violations, excluding them would undercount linkages. Therefore, we assigned them a weight of 0.5.^3^ Fourth, following the classification of 16 polluting industries published by MEE in 2008 (https://www.mee.gov.cn/gkml/hbb/bgth/200910/t20091022_174891.htm), we divided the listed firms into MEE-listed polluting and other categories. We refer to listed firms in MEE-listed polluting industries as polluting listed firms for brevity in the subsequent text. Fifth, after excluding data on intra-city penalties and penalty decisions issued by provincial and higher-level administrative authorities, 1,843 relevant records remain. Using cleaned data, we constructed two types of cross-regional environmental violation networks in China: (1) the network of polluting listed firms and (2) the network of other listed firms.

To objectively capture the spatiotemporal evolution of cross-regional environmental violations in China and to avoid abrupt fluctuations caused by special events in a single year, we adopted a segmented-period approach. In particular, the implementation of the *Environmental Protection Tax Law of the People’s Republic of China* in 2018 marked a significant policy showing greater reliance on economic measures and market mechanisms. Penalty volumes exhibited a moderate decline after 2018, followed by stabilization and a slight increase during 2020–2021. This pattern is temporally consistent with the implementation of the 2018 policy but does not indicate a causal linkage. To avoid short-term data instability caused by an overly long statistical cycle, we did not incorporate penalty data for 2024 and 2025 in our analysis. Accordingly, we divided the study period into two subperiods: 2014–2018 and 2019–2023.

#### Social network analysis

SNA is a mature quantitative method that focuses on the relational structure between actors, which can effectively reveal the overall structural characteristics of intercity linkages, the core-edge structure of the network, and the role positioning of city nodes.^40^^,^^58^ This method has been widely and successfully applied in the research on spatial association networks of pollution, carbon emissions, and energy consumption in existing studies.^45^^,^^59^ Compared with traditional non-relational statistical methods, SNA is highly consistent with the research objectives of this study.

Using Gephi software, we analyzed the average path length, network density, clustering coefficient, and node-weighted degree centrality of the cross-regional environmental violation networks to examine their fundamental properties and spatial distribution patterns.^38^^,^^40^^,^^41^^,^^57^^,^^60^^,^^61^ In the network, edges represent the sum of weighted penalty values for a specific city pair (A→B). Multiple penalty records of the same firm for the same city pair are aggregated into a single edge weight. This is to avoid double-counting and accurately reflect the overall violation intensity between the two cities. Nodes represent the cities where firms are registered and where penalty records occur.

At the network level, indicators including the number of nodes, the number of edges, network density, average degree, average weighted degree, network diameter, average clustering coefficient, and average path length were calculated to evaluate the connectivity and organizational characteristics of the networks. Changes in these indicators over time were used to identify the dynamic evolution of cross-regional pollution transfer linkages.

$$Density=\frac{Edges}{N\left(N-1\right)}$$

*N* represents the number of nodes in the network. The higher the network density, the closer the cross-regional environmental risk links between cities.

$$Average\;degree=\frac{Edges}{N}$$

$$Average\;weighted\;degree\;\left(s\right)=\frac{\sum_{i=1}^{N}s_{i}}{N}$$

$$s_{i}=\sum_{j=1}^{N}w_{ij}$$

*s*_*i*_represents the weighted degree of node *i*, and *w*_*ij*_ represents the weight of the edge between node *i* and node *j*. The average weighted degree indicates the average level of node connection strength in the network.

$$\text{Clustering}\;\text{Coefficient}\;\left(C_{i}\right)=\frac{e_{i}}{d_{i}\left(d_{i}-1\right)}$$

*e*_*i*_ represents the actual number of connections among neighbors of node *i*. *d*_*i*_ represents the degree of node *i*. A higher clustering coefficient indicates that the network tends to form stronger local agglomeration structures.

$$Average\;Path\;Length\;\left(L\right)=\frac{1}{N\left(N-1\right)}\sum_{i\neq j}^{N}d_{ij}$$

*d*_*ij*_ denotes the shortest path length between node *i* and node *j*. A smaller average path length indicates higher efficiency of network transmission.

At the node level, degree centrality was used to identify core cities within the networks. Visualization techniques were further applied to display the spatial distribution and linkage intensity of intercity environmental penalty relationships.

$$C_{D}\left(i\right)=\frac{d_{i}}{N}$$

*C*_*D*_(*i*)represents the degree centrality, which measures the central position of a node in the network.

Comparative analyses were conducted between polluting industry firms and other industry firms to explore differences in network expansion, connectivity, and spatial dependence patterns.

#### Spatial statistical method

By employing Global Moran’s I and Getis-Ord Gi∗ tools in ArcGIS 10.8 software, we investigated the spatial clustering characteristics of cross-regional environmental violations.^62^^,^^63^^,^^64^ A Queen contiguity spatial weight matrix was used to define spatial adjacency, and 999 permutations were applied for significance testing. Hot and cold spots were classified into four levels: cold spot, sub-cold spot, sub-hot spot, and hot spot, using the natural breakpoint (Jenks) method.

#### Geodetector

With Geodetector software, we explored the driving mechanisms of cross-regional environmental violations by examining the relationships between cities’ weighted degree centrality, weighted in-degree, and out-degree, and factors such as economic development level, industrial structure, and environmental regulation.^58^^,^^59^^,^^65^^,^^66^ The explanatory power of each factor is measured using the q-statistic, assessed using 999 random permutations, which quantifies the proportion of spatial variance in the dependent variable that can be explained by the stratification of the explanatory variable. Unlike multiple linear regression models, the q-statistic, calculated independently for each factor, measures the proportion of spatial variance in the dependent variable explained by that factor alone.^67^ The correlation between explanatory variables will not lead to bias in the q-statistic measurement.

#### Construction of weighted intercity networks

Based on the identified off-site environmental penalty records, weighted directed intercity environmental networks were constructed. In the networks, cities represent nodes, while off-site environmental penalty flows between cities represent directed edges. Specifically, a directed linkage from city *i* to city *j* indicates that a listed firm registered in city *i* received an environmental penalty in city *j*.

The weight of each edge corresponds to the frequency of off-site environmental penalty events between two cities during a given year. Separate intercity networks were constructed for polluting industry firms and other industry firms to compare their structural evolution characteristics.

The resulting adjacency matrices were analyzed using social network analysis methods to reveal the spatial organization, connectivity patterns, and evolution trajectories of cross-regional environmental risks in China.

### Quantification and statistical analysis

Descriptive statistics were used to summarize the characteristics of environmental penalties and network structures. Temporal trends and spatial evolution characteristics were analyzed through comparative network indicators and visualization methods. Statistical significance in the geographical detector analysis was evaluated according to conventional significance thresholds. All figures and network diagrams were generated based on processed intercity linkage matrices.

### Additional resources

This research did not utilize any additional resources.
